# Supplementary material for: Assessing the Intense Influenza A(H1N1)pdm09 Epidemic and Vaccine Effectiveness in the Post-COVID Season in the Russian Federation
Source: Viruses. 2023 Aug 21;15(8):1780. doi: 10.3390/v15081780 (PMC10458445; doi:10.3390/v15081780)
Supplement: Supplementary file 1 [file viruses-15-01780-s001.zip › Titles for figures.pdf]

Titles for figures

Supplementary Figure S1. Geography of influenza A(H1N1) pdm09, A(H3N2) and B viruses spread in Russia, season 2022-2023.

Supplementary Figure S2. Comparative data on etiology of the contemporary influenza epidemics in the Russian Federation.

Supplementary Figure S3. 3D antigenic map of influenza viruses A(H3N2) isolated in Russia in the epidemic season 2022-2023.
Note: designations as in figure 4; blue circles – WHO recommended vaccine strains for the season 2022-2023.

Supplementary Figure S4. Phylogenetic comparison of HA genes of influenza A(H3N2) viruses (ML tree, GTR+GAMMA, constructed with RaxML).
