# Supplementary figures and images for: Assessing the Intense Influenza A(H1N1)pdm09 Epidemic and Vaccine Effectiveness in the Post-COVID Season in the Russian Federation
Source: Viruses. 2023 Aug 21;15(8):1780. doi: 10.3390/v15081780 (PMC10458445; doi:10.3390/v15081780)

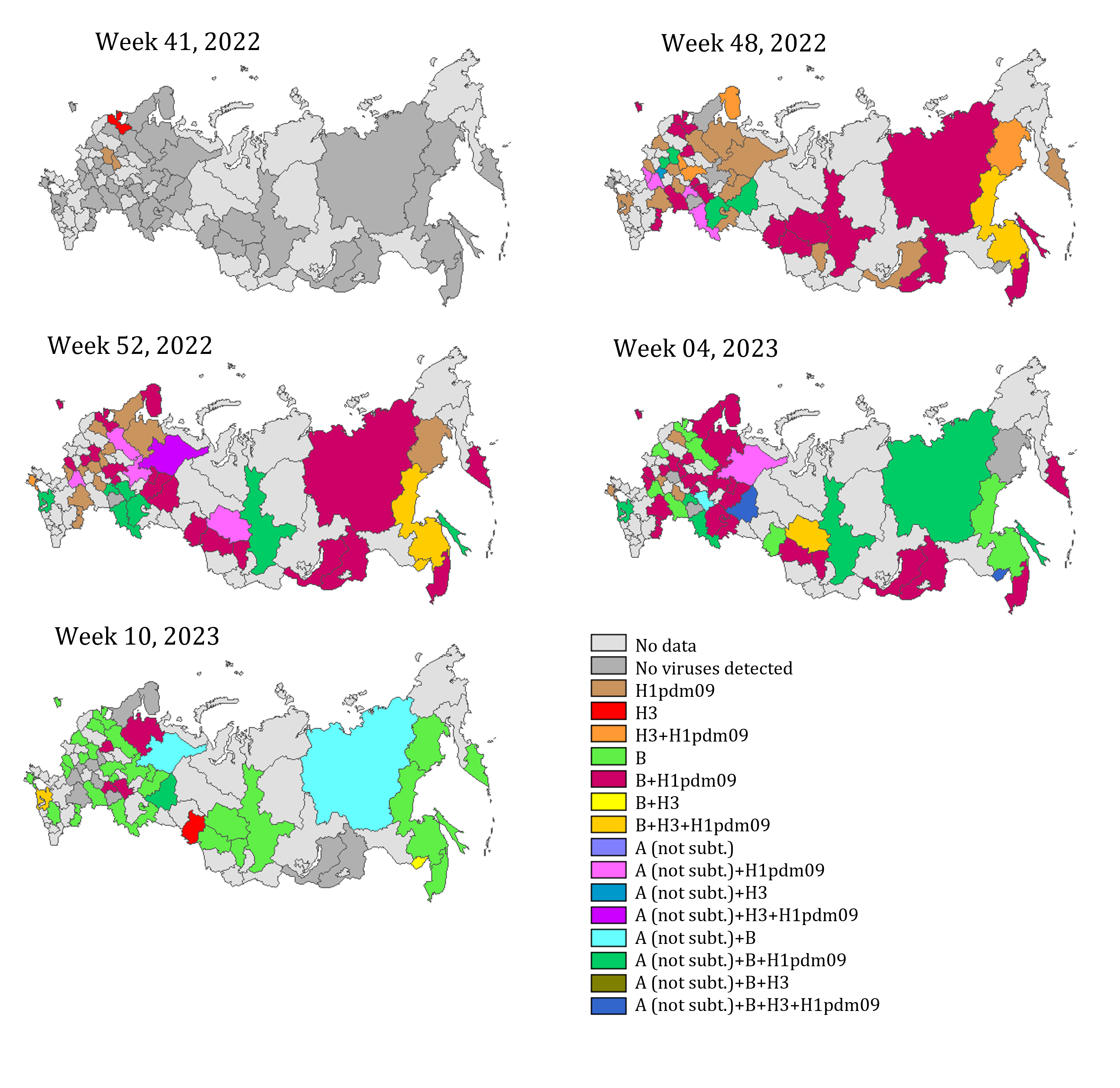

Supplement: Supplementary file 1 [file viruses-15-01780-s001.zip › Supplementary figure S1.tif]

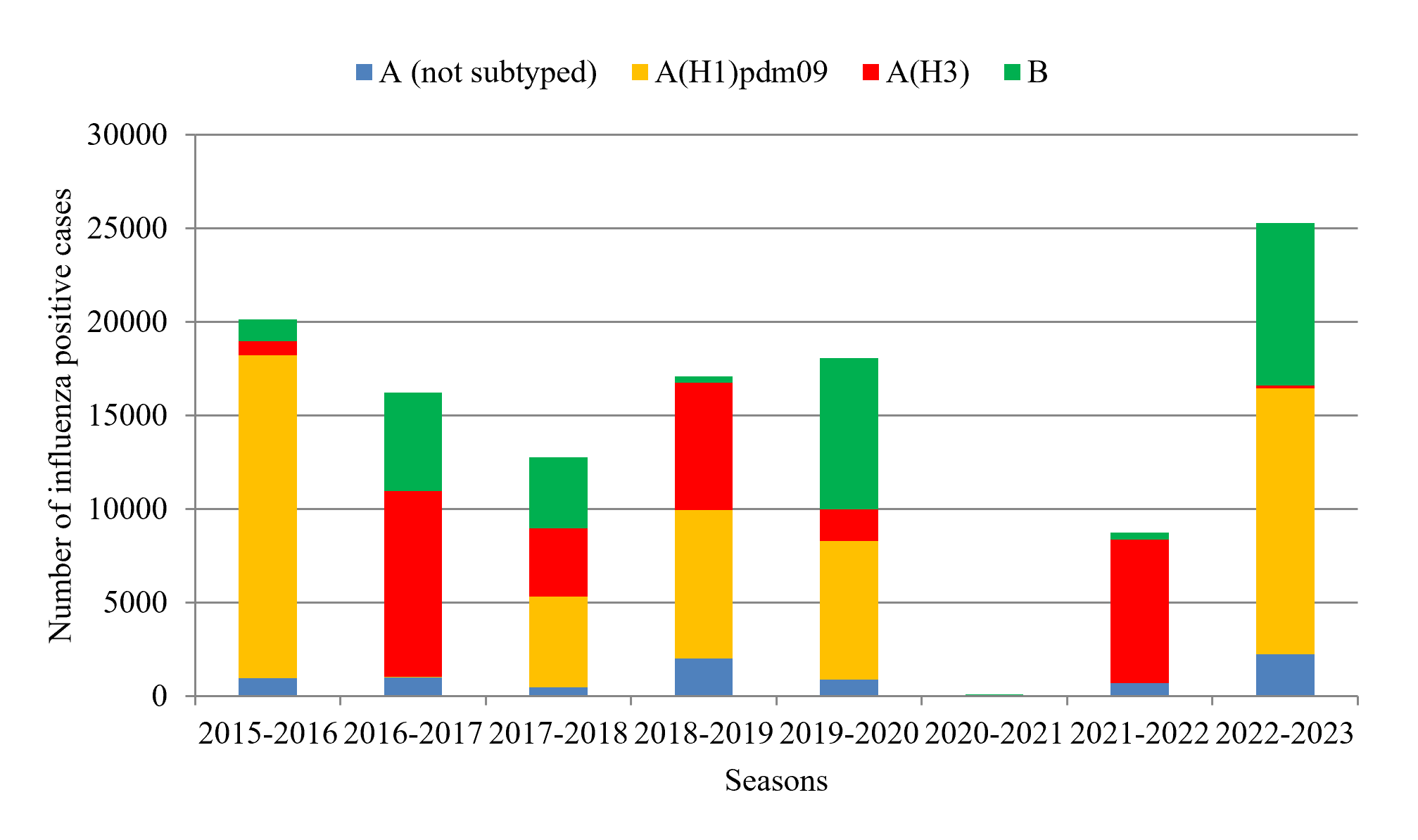

Supplement: Supplementary file 1 [file viruses-15-01780-s001.zip › Supplementary figure S2.tif]

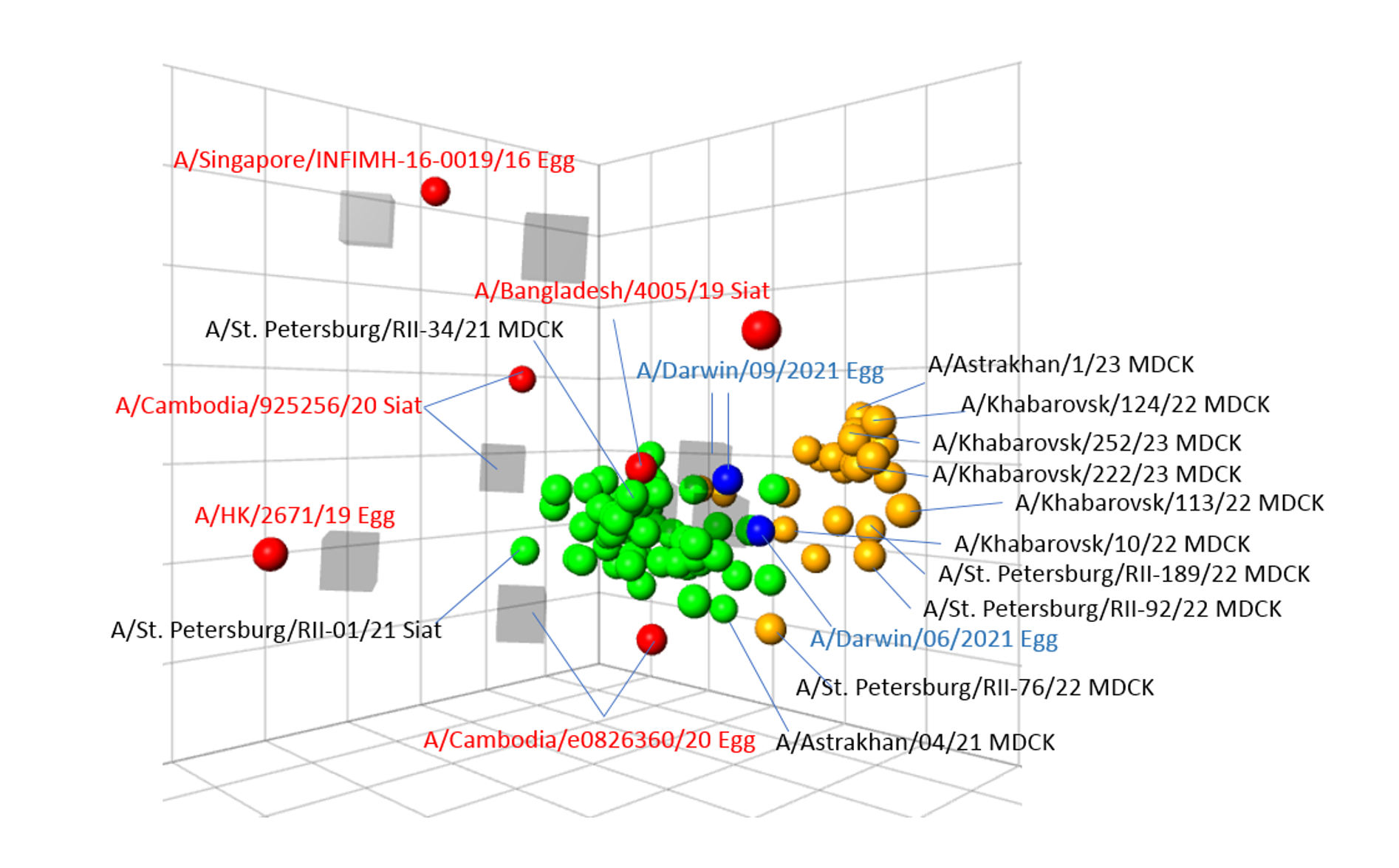

Supplement: Supplementary file 1 [file viruses-15-01780-s001.zip › Supplementary figure S3.png]

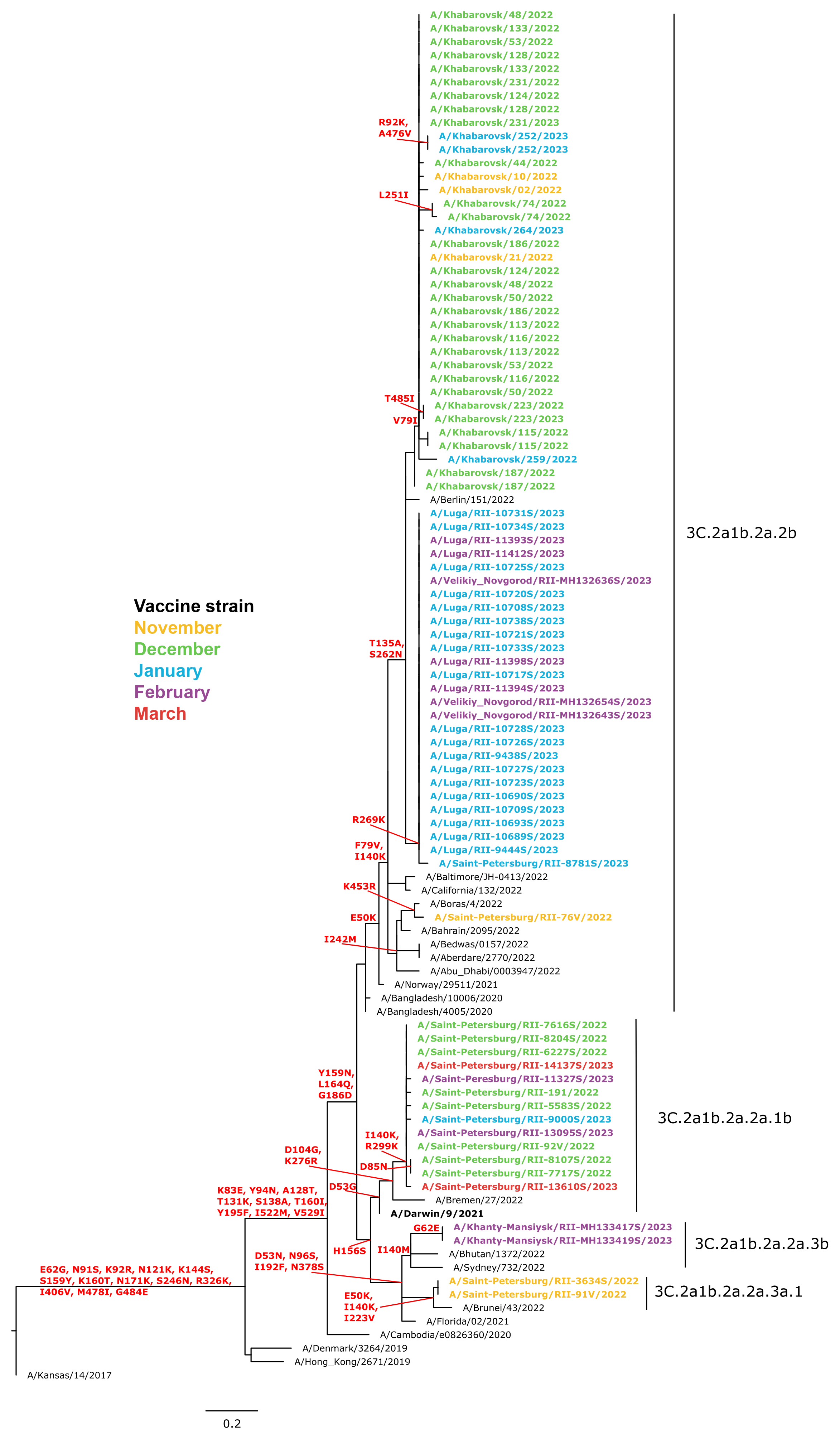

Supplement: Supplementary file 1 [file viruses-15-01780-s001.zip › Supplementary figure S4.png]
